# Systemic Mastocytosis Associated with Splenic Marginal Zone Lymphoma with Villous Lymphocytes

**DOI:** 10.1155/2011/385074

**Published:** 2011-07-11

**Authors:** R. Fernández-Torres, M. M. Verea, A. Álvarez, P. Torres, E. Fonseca

**Affiliations:** ^1^Departments of Dermatology, Complejo Hospitalario Universitario A Coruña, Sir John Moore S/N, 15001 La Coruña, Spain; ^2^Departments of Pathology, Complejo Hospitalario Universitario A Coruña, Sir John Moore S/N, 15001 La Coruña, Spain; ^3^Departments of Haematology, Complejo Hospitalario Universitario A Coruña, Sir John Moore S/N, 15001 La Coruña, Spain

## Abstract

The development of a second haematological disease during the course of systemic mastocytosis is a well-known phenomenon. In most of the cases, they consist of myelodysplasia or myeloproliferative disorders. The association with lymphoproliferative disorders has also been described, but it is uncommon and the relationship is not well established. We report a patient diagnosed with systemic mastocytosis who three years later developed a splenic marginal zone lymphoma with villous lymphocytes.

## 1. Introduction

The term mastocytosis refers to a group of disorders characterized by and abnormal growth and accumulation of mast cells in one or more organs, including skin, lymph nodes, bone marrow, nervous system, gastrointestinal tract, spleen, and liver. 

Systemic mastocytosis associated with clonal haematological nonmast cell lineage disease (SM-AHNMD) is an entity recognized by the World Health Organization classification of tumours of hematopoietic tissues [1].

The occurrence of myeloproliferative syndromes in patients with systemic mastocytosis is a well-recognized phenomenon. However, non-Hodgkin's lymphoma is very uncommon.

We report a patient with urticaria pigmetosa of ten years evolution and systemic mastocytosis (SM) diagnosed three years ago who developed splenic marginal zone lymphoma with villous lymphocytes, a low-grade B-cell non-Hodgkin's lymphoma characterized by splenomegaly, moderate lymphocytosis, bone marrow infiltration, serum monoclonal band, and a benign course with response to splenectomy [2]. To our knowledge, the association of this neoplasm with SM has not been previously documented in the literature.

## 2. Case Report

**Figure 1:**
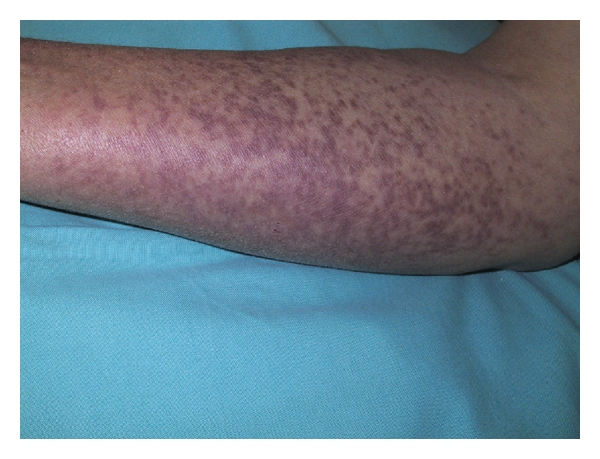
Papular, purplish cutaneous lesions of urticaria pigmentosa on the forearms.

A 65-year-old female with unremarkable past medical history presented with papular, purplish, and itchy cutaneous lesions located on her forearms, thighs, and trunk for ten years (Figure 1). 

Laboratory investigations, including blood counts, chemistry, and routine coagulation parameters, showed no alterations. A skin biopsy from a papular lesion revealed an acanthotic epidermis and hyperpigmentation of the epidermal basal cell layer. Giemsa stain showed many mast cells around blood vessels and scattered throughout the dermis.

A bone marrow smear showed atypical large, spindle-shaped, and hypogranulated mast cells with elongated nucleus. The histological examination of a bone marrow biopsy disclosed decreased cellularity and multifocal infiltrates of cells with oval nucleus and large cytoplasm. These cells were positive with the monoclonal mast cell stain and located surrounding blood vessels or with interstitial distribution. Surrounding these cells, small lymphocytic aggregates were observed. 

According to the cutaneous findings and the bone marrow infiltration, the diagnosis of systemic mastocytosis was made. 

Because of a moderate pruritus, the patient was treated with H_1_-receptor blocking agents.

Three years later, a routine laboratory examination showed anaemia, lymphocytosis, increased *β*2-microglobulin (2.54 mg/l, normal value: 1.5–2.3), and a serum monoclonal band of lambda light chains. Physical examination showed hepatosplenomegaly and lymphadenopathy, which was confirmed by an abdominal echography. 

**Figure 2:**
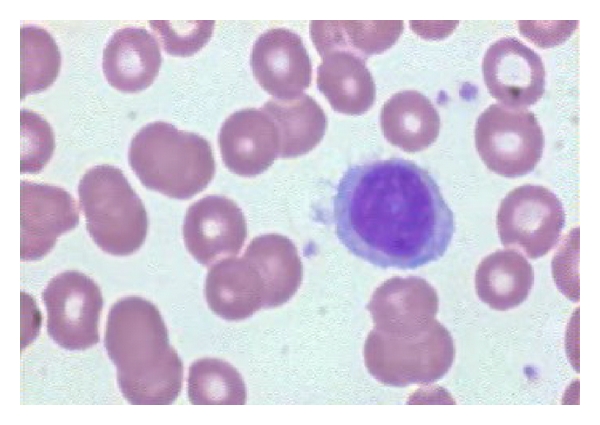
Peripheral blood smear showing an atypical lymphocyte with elongated cytoplasm processes.

Peripheral blood smear showed morphologically abnormal lymphocytes with elongated cytoplasmic extensions (Figure 2). Immunohistochemical studies disclosed a B immunophenotype characterized by the expression of CD22, FMC7, CD20, and slight expression of CD11c, CD10, CD38, and negative for CD5 and CD23.

**Figure 3:**
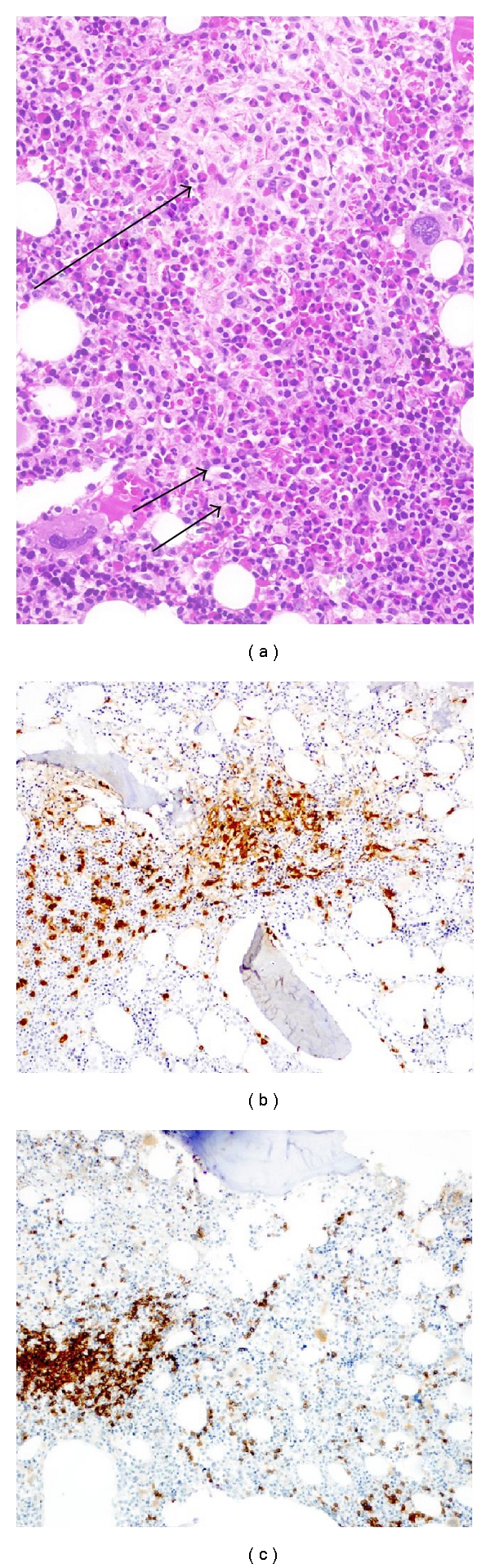
(a) Bone marrow biopsy with areas of infiltration by villous lymphocytes (2 arrows) and areas of infiltration by mast cells and fibrosis (arrow) (haematoxylin-eosin, original magnification ×200). (b) Areas of mast cell infiltration in the bone marrow (Mast cell stain ×150). (c) Villous lymphocytes in the bone marrow (CD20 stain ×150).

A new bone marrow biopsy showed nodular infiltration by atypical mast cells and nodular interstitial infiltration by lymphoid cells positive with CD 20 stain (Figure 3). 

These features were consistent with the diagnosis of splenic marginal zone lymphoma with villous lymphocytes. Thus, the diagnosis of systemic mastocytosis with coexisting splenic marginal zone lymphoma with villous lymphocytes was established. 

She received chemotherapy with chlorambucil for 28 months with progressive improvement of laboratory test and parallel clearing of trunk cutaneous lesions, but not on the forearms and thighs. Two new skin biopsies of persistent lesions and cleared skin showed similar number of mast cells in both.

## 3. Discussion

A significant number of patients with systemic mastocytosis (5–40%) may develop an associated haematological clonal nonmast cell lineage disease (SM-AHNMD). Both neoplasms are usually synchronously diagnosed on the basis of morphological evaluation of a bone marrow biopsy, although later development of AHNMD in patients with long standing mastocytosis, as in our patient, is also possible [3]. 

Any type of haematological malignancy may be associated with SM. However, myeloid neoplasms develop with a much higher frequency than lymphoid neoplasms [4, 5]. Among 138 cases of SM-AHNMD reported by Pardanani et al., 123 (89%) had associated myeloid neoplasm and only 7 (5%) had lymphoma [6].

The pathophysiologic basis of simultaneous occurrence of SM and other neoplasms is not completely understood. In the case of myeloid neoplasms, the first possibility is that a hematopoietic progenitor cell, capable of given rise to mast cells and other myeloid cells, is involved. In this case, two clinically distinct disorders develop due to subclone formation [7]. It has also been suggested that mast cells may produce growth factors capable of stimulating the proliferation of other cell lines [8, 9].

The codevelopment of SM and lymphoid malignancies, due to the small number of cases reported, may be incidental or may occur as a result of genetic instability. 

Kim et al. demonstrated distinct clonal origins in mastocytosis and associated B-cell lymphomas [10]. However, there is evidence suggesting that B cells share a common progenitor with macrophages, a cell lineage that appears to be closely related to mastocytes, and c-Kit mutations have been detected in B lymphocytes, apart from neoplastic mastocytes, so the same origin of both neoplasm is another possibility [11].

Patients with SM and lymphoproliferative diseases usually have a long history of indolent SM, which suggests a different pathogenesis for the two entities. In our case, NHL developed three years after the diagnosis of SM in a patient with urticaria pigmentosa of ten years evolution.

Treatment and prognosis of SM-AHNMD depend on the associated haematological disease. Chemotherapy usually affects the associated haematological neoplasm with little or no effect on mast cell disease [12, 13]. In our patient, improvement of cutaneous lesions on the trunk was observed after treatment with chlorambucil, but histological examination showed no decrease in the number of mast cells. The significance of this fact is not well understood, and it might be associated with a poor prognosis in patients with associated hematologic disorders [14]. It is believed that it may be a natural process in the evolution of SM or could be related with a change in homing receptor mast cells.

## 4. Conclusion

In summary, we report a case of SM-AHNMD, with the peculiarity that the associated neoplasm was a splenic marginal zone lymphoma, an association of which we have found no previous reports in the literature.
